# Complex organisation and structure of the ghrelin antisense strand gene GHRLOS, a candidate non-coding RNA gene

**DOI:** 10.1186/1471-2199-9-95

**Published:** 2008-10-28

**Authors:** Inge Seim, Shea L Carter, Adrian C Herington, Lisa K Chopin

**Affiliations:** 1 Institute of Health and Biomedical Innovation, Queensland University of Technology, Kelvin Grove, Queensland, Australia

## Abstract

**Background:**

The peptide hormone ghrelin has many important physiological and pathophysiological roles, including the stimulation of growth hormone (GH) release, appetite regulation, gut motility and proliferation of cancer cells. We previously identified a gene on the opposite strand of the ghrelin gene, ghrelinOS (*GHRLOS*), which spans the promoter and untranslated regions of the ghrelin gene (*GHRL*). Here we further characterise *GHRLOS*.

**Results:**

We have described *GHRLOS *mRNA isoforms that extend over 1.4 kb of the promoter region and 106 nucleotides of exon 4 of the ghrelin gene, *GHRL*. These *GHRLOS *transcripts initiate 4.8 kb downstream of the terminal exon 4 of *GHRL *and are present in the 3' untranslated exon of the adjacent gene *TATDN2 *(TatD DNase domain containing 2). Interestingly, we have also identified a putative non-coding *TATDN2-GHRLOS *chimaeric transcript, indicating that *GHRLOS *RNA biogenesis is extremely complex. Moreover, we have discovered that the 3' region of *GHRLOS *is also antisense, in a tail-to-tail fashion to a novel terminal exon of the neighbouring *SEC13 *gene, which is important in protein transport. Sequence analyses revealed that *GHRLOS *is riddled with stop codons, and that there is little nucleotide and amino-acid sequence conservation of the *GHRLOS *gene between vertebrates. The gene spans 44 kb on 3p25.3, is extensively spliced and harbours multiple variable exons. We have also investigated the expression of *GHRLOS *and found evidence of differential tissue expression. It is highly expressed in tissues which are emerging as major sites of non-coding RNA expression (the thymus, brain, and testis), as well as in the ovary and uterus. In contrast, very low levels were found in the stomach where sense, *GHRL *derived RNAs are highly expressed.

**Conclusion:**

*GHRLOS *RNA transcripts display several distinctive features of non-coding (ncRNA) genes, including 5' capping, polyadenylation, extensive splicing and short open reading frames. The gene is also non-conserved, with differential and tissue-restricted expression. The overlapping genomic arrangement of *GHRLOS *with the ghrelin gene indicates that it is likely to have interesting regulatory and functional roles in the ghrelin axis.

## Background

Ghrelin, a hormone with many physiological and pathophysiological roles, was initially described as the endogenous ligand for the growth hormone secretagogue receptor (GHSR 1a), through which it stimulates the release of growth hormone from the anterior pituitary [1]. Ghrelin is primarily produced in the stomach and plays a key role in regulating appetite, gut motility and energy balance [2-6]. Ghrelin is also an autocrine factor in a number of tissues, as it regulates insulin release and has therapeutic potential for inflammatory diseases, heart disease, cancer cachexia, diabetes mellitus and obesity [7]. Despite the importance of ghrelin in a range of physiological systems and pathophysiological conditions, little is known about the regulation of ghrelin synthesis and secretion. We previously identified a gene on the opposite strand of the ghrelin gene, ghrelinOS (*GHRLOS*), which spans the promoter and untranslated regions of the ghrelin gene (*GHRL*) [8]. However, the genomic structure, expression pattern and potential function of *GHRLOS *remains to be investigated. It is not known whether *GHRLOS *RNA species with open reading frames exist, or whether *GHRLOS *is a non-coding RNA gene.

There is strong support for the hypothesis that antisense transcripts provide a widespread and important mechanism for the regulation of the human genome [9,10]. Our understanding of the genome is currently undergoing a paradigm shift, as a previously hidden and complex layer of antisense and non-coding RNAs is emerging, which controls gene transcription and translation through a diverse range of mechanisms [11]. Much phenotypic diversity between humans and other species is likely to be due to regulation by RNA [12].

In this study, we examined the genomic structure and organisation of *GHRLOS*. We have found that *GHRLOS *spans approximately 44 kb of genomic DNA and transcribes long, 5' capped, polyadenlyated RNA species that are extensively spliced and differentially expressed. High levels of *GHRLOS *expression occur in the emerging non-coding RNA tissues, the brain, testis and thymus. We have also examined *GHRLOS *RNA species *in silico*, revealing that *GHRLOS *is a candidate non-coding RNA gene. These data provide a strong basis for further functional studies to determine whether *GHRLOS *plays a role in the regulation of ghrelin gene expression.

## Results

### Characterisation of *GHRLOS* start sites and alternative splicing

**Figure 1:**
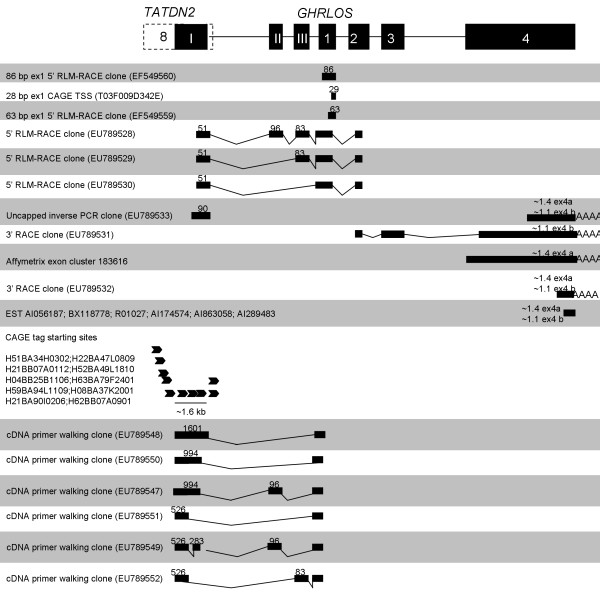
**Mapping of *GHRLOS *transcript 5' and 3' ends**. Relative positions of the 5' and 3' ends of *GHRLOS *transcripts were derived from RACE, inverse PCR and cDNA primer walking RT-PCR experiments, as well as from publically available data from EST databases, Affymetrix exon arrays and CAGE tags. *TATDN2 *exons are shown as white boxes, *GHRLOS *exons as black boxes, introns as horizontal lines. Size (bp) of selected exons are shown above each exon. All known 3' ends of *GHRLOS *transcripts contain the same polyadenylation signal. Poly(A) tails are represented by AAAA. Cap-analysis gene expression (CAGE) tags are indicated by arrows, and CAGE-aided cDNA primer walking amplicons are displayed below the arrows. Not drawn to scale.

An initial aim of this study was to characterise *GHRLOS *in a range of tissues. As we have previously demonstrated the expression of *GHRLOS *mRNA transcripts in the human stomach [8], we performed 5' RLM-RACE (RNA ligase mediated rapid amplification of cDNA ends) on this tissue. Unexpectedly, we identified a number of new exons (exon I-III) [GenBank:EU789528, EU789529, and EU789530] that are 2.5 to 4.8 kb upstream of the previously reported *GHRLOS *transcription start sites [8] in exon 4* (4*a-c). To simplify the numbering of GHRLOS exons, the previously reported [8] exons 4*, 2**, 2* and -1* have been renamed exon 1 to 4, respectively; while the exons upstream of the reported start sites in exon 1 are denoted by Roman numerals (I-III). Importantly, in the *GHRLOS *variants demonstrated via 5' RLM-RACE, exon 1 is extended, with a 106 nt region (exon 1d, hereafter termed exon 1) overlapping exon 4 of the ghrelin gene (see [Additional file 1]). The novel first exon, exon I, is 51 bp in size (Fig. 1) and overlaps the 3' untranslated region of the adjacent gene, *TATDN2 *(TatD DNase domain containing 2, also known as hypothetical protein KIAA0218) [13]. This gene initiates on the same DNA strand as *GHRLOS*, approximately 32,000 base pairs upstream of the 51 bp exon I of *GHRLOS*.

In order to determine the polyadenylation site(s) of *GHRLOS *transcripts, 3' RACE and inverse PCR were conducted using normal stomach and prostate tissues, the RWPE-1 prostate cell line and the PC3 prostate cancer cell line (which is derived from prostate cancer cells that have metastasised to the bone). We obtained 3' RACE clones from the PC3 prostate cancer cell line, which demonstrated a 1.4 kb exon 4a and a 1.1 kb exon 4b, with the putative polyadenylation signal AAATTA [GenBank:EU789531, and EU789532] (Fig. 1). The 3' RACE data matches a number of Expressed Sequence Tags (ESTs) from subchondral bone [GenBank:BM991802], foetal liver [GenBank:AI056187], pooled liver and spleen [GenBank:BX118778; R01027], Wilms' tumour (a paediatric cancer of the kidney) [GenBank:AI174574], pooled brain cancers [GenBank:AI863058] and high grade serous carcinoma of the uterus [GenBank:AI289483]. Moreover, we performed uncapped inverse PCR, and sequencing of amplicons from the PC3 cell line revealed a 90 bp exon I and exon 4 sequence with a 3' end differing by less than 20 base pairs when compared to the 3' RACE and reported ESTs [GenBank:EU789533]. Interestingly, the putative 3' end of exon 4 corresponds to publicly available Affymetrix Human Exon 1.0 ST array data (Exon Cluster ID 183613). Furthermore, all of the amplicons that we obtained were followed by a stretch of adenosines at the 3' ends that are not present in the genomic sequence. We, therefore, concluded that we had reached a genuine 3' end of *GHRLOS*.

### Complex pattern of *GHRLOS* variant exon expression

**Figure 2:**
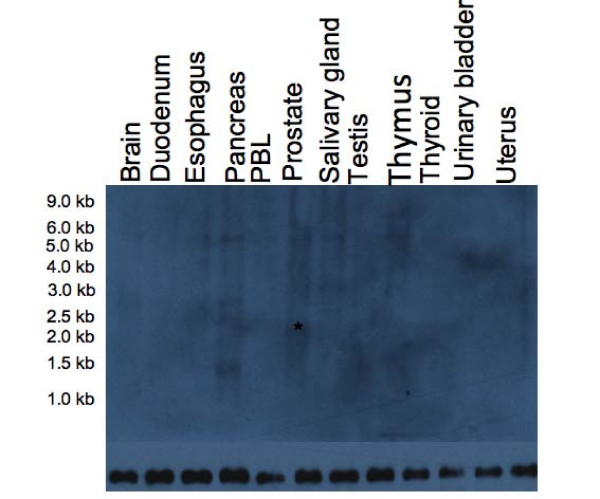
**Northern blot of poly(A)^+ ^RNA from normal, human tissues with strand-specific, DIG-labelled riboprobe**. Northern blot of 2 μg poly(A)^+ ^RNA from 12 normal tissues (OriGene) probed for exon 1, which is an exon common to all known *GHRLOS *mRNA isoforms (top panel). The position of the RNA size markers is indicated (RNA Millennium Markers, OriGene). A pound mark (#) indicates a putative 1.7–1.8 kb (approximately 2.0 kb with polyadenylation tail) full-length transcript in the prostate, as deduced from RLM-RACE and RT-PCR experiments. Several other transcripts 1–5.5 kb in size could be seen. The same membrane was stripped and hybridized with a β-actin cRNA probe as a loading control (bottom panel).

In order to examine the size and tissue distribution of *GHRLOS *transcripts, we performed Northern blot analysis of mRNA from human stomach and 12 normal, human tissues. With a riboprobe designed to span exons I, II, 1 and 2 of *GHRLOS*, a weak, smeared signal ranging from approximately 1.0 to 2.0 kb in size was observed in the human stomach upon a lengthened exposure time (data not shown). A riboprobe spanning exon 1 alone (a region common to all *GHRLOS *RNA isoforms) resulted in signals ranging from 1.0 to 5.5 kb in size in the pancreas, prostate, salivary gland and thymus (Fig. 2). Upon longer exposure times, a smear in the 1.0 to 5.5 kb range was seen in all tissues, except for peripheral blood leukocytes and urinary bladder (data not shown). This suggests that *GHRLOS *is a fully processed transcript consisting of many mRNA isoforms.

### *GHRLOS* exons are highly polymorphic

**Figure 3:**
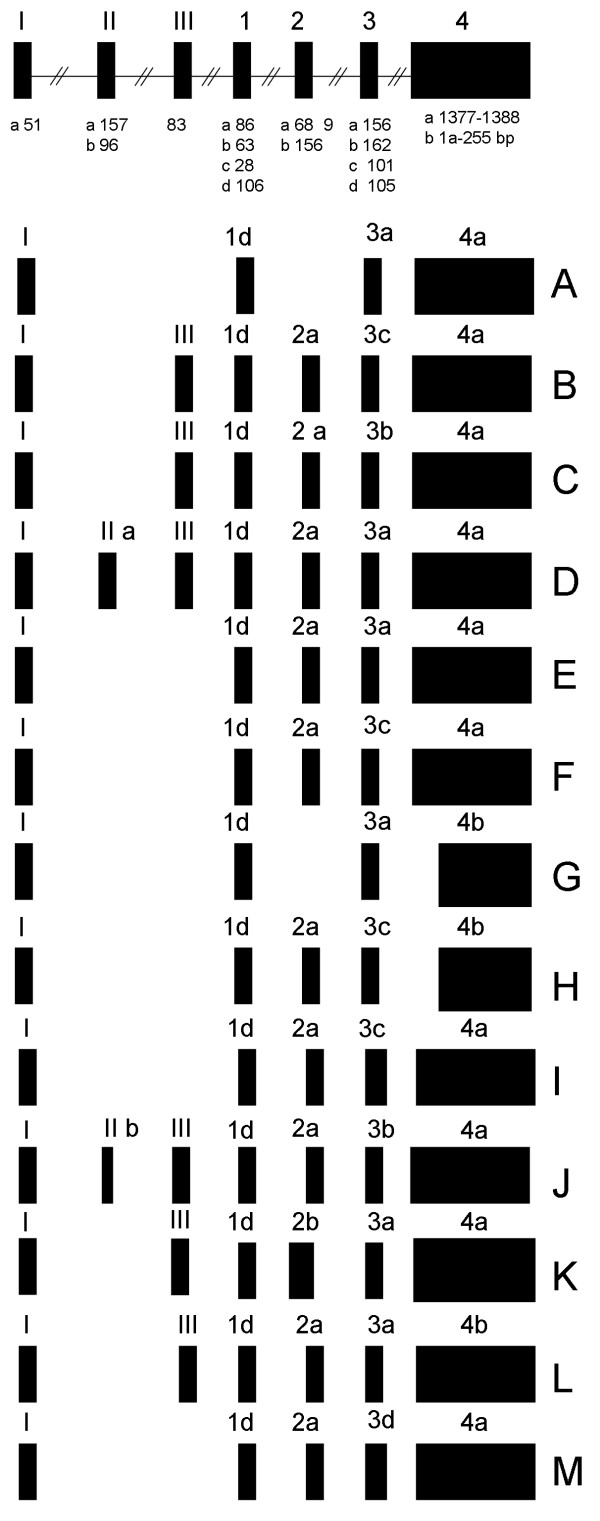
**Complex pattern of GHRLOS variant exon expression**. *GHRLOS *transcripts A to M were demonstrated by RT-PCR, while the 5' end (exon I) and 3' end (exon 4) were determined by RACE, inverse PCR and by examining publically available Affymetrix exon array data and expressed sequence tag (EST) data. Exons are represented as boxes, introns as horizontal lines and sizes (bp) are indicated below each exon. The size of exon 4a is listed as 1377–1388 and corresponds to a transcript which is polyadenylated 20–30 bp from the same polyadenylation signal (data not shown). Exon 4b harbours the same polyadenlation site, but is 5' truncated (250 bp). GhrelinOS variants A to M have been deposited to GenBank: A-F, stomach [GenBank:EU789534, EU789535, EU789536, EU789537, EU789538, and EU789539]; G-H, prostate [GenBank:EU789540, and EU789541]; I, foetal brain [GenBank:EU789542]; J-M, heart [GenBank:EU789543, EU789544, EU789545, and EU789546].

The RACE, inverse RT-PCR and Northern blotting experiments indicate that *GHRLOS *is extensively spliced and that isoforms range greatly in size (from approximately 1.0–5.5 kb). To examine the alternative splicing pattern of *GHRLOS *in greater detail, we performed RT-PCR using a range of human tissues and cell lines. We used a forward primer common to exon Ia (identified via 5' RLM-RACE) and a reverse primer in a region common to exons 4a and b. Sequence analysis indicated that, with the exception of exons 1 and 4, which are common to all known *GHRLOS *isoforms, *GHRLOS *exons are highly polymorphic in size and exon skipping occurs frequently. A representative amplicon banding pattern is shown in [Additional File 2]. The highly variable splicing pattern revealed by RT-PCR is consistent with the diffuse and broad signal which we detected by Northern blotting. In total we obtained 13 different *GHRLOS *splice variants (Fig. 3). Analysis using GMAP, a genomic mapping and alignment program for mRNA and EST sequences [14], indicated that all *GHRLOS *exons are flanked by canonical splice donor and acceptor sites (GT/AG), except for exons 3a and 3c where the splice junction is the common non-canonical splice pair GC/AG [15]. *GHRLOS *exons and introns identified are listed in [Additional File 3].

### Extending the known length of *GHRLOS*

Northern blotting demonstrated *GHRLOS *transcripts 1–5.5 kb in size, however, further RT-PCR and 3' RACE analysis with a number of different primer combinations failed to reveal *GHRLOS *transcripts larger than 2 kb (data not shown). For this reason, we hypothesised that the ghrelin antisense gene may extend further upstream. Capped RLM-RACE of stomach tissue and uncapped inverse PCR of the PC3 prostate cancer cell line (Fig. 1) indicated that a *GHRLOS *promoter (exon I) was present at the very end of the 2 kb 3' untranslated exon 8 of *TATDN2*. Moreover, we discovered that multiple Cap Analysis of Gene Expression (CAGE) tags are clustered approximately 1.5 kb upstream of the *GHRLOS *transcription start sites found via 5' RLM-RACE. CAGE tags are an average of 20–21 nucleotides and are produced by large-scale sequencing of concatemers derived from the 5' ends of capped mRNA [16,17]. The CAGE method, therefore, detects the most 5' site of the mRNA transcripts (the transcription start site) and gives an unbiased and comprehensive picture of the positions and usage of transcription start sites [18]. To confirm if this region belongs to *GHRLOS*, we employed nested RT-PCR using thymus tissue, foetal brain tissue and Hep G2 hepatocarcinoma cell line cDNA (with RNA reverse transcribed using oligo(dT) primers). The forward primers were present immediately downstream of the CAGE tag cluster (which is 1.5 kb upstream of the 51 bp exon I of *GHRLOS*) and the reverse primers spanned exon 1 (which is common to all known *GHRLOS *variants). Sequence analysis revealed several novel exon I variants, which were approximately 1601 bp, 994 bp and 526 bp in length. All of these variants spliced into the expected acceptor site of the 106 bp exon 1 (Fig. 1 and [Additional File 4]). The end of the 1601 bp exon I (termed exon Ib) spanned the 51 bp exon I (exon Ia) identified via capped RLM-RACE of the stomach, while the other exons (Ic-d) employed different upstream donor sites, suggesting that they are alternative first exons. The data demonstrate that there are at least two first *GHRLOS *exon regions in the untranslated region of *TATDN2*. This suggests that the *GHRLOS *promoter is a broad-type, TATA-less promoter that initiates transcription at many sites [19].

**Figure 4:**
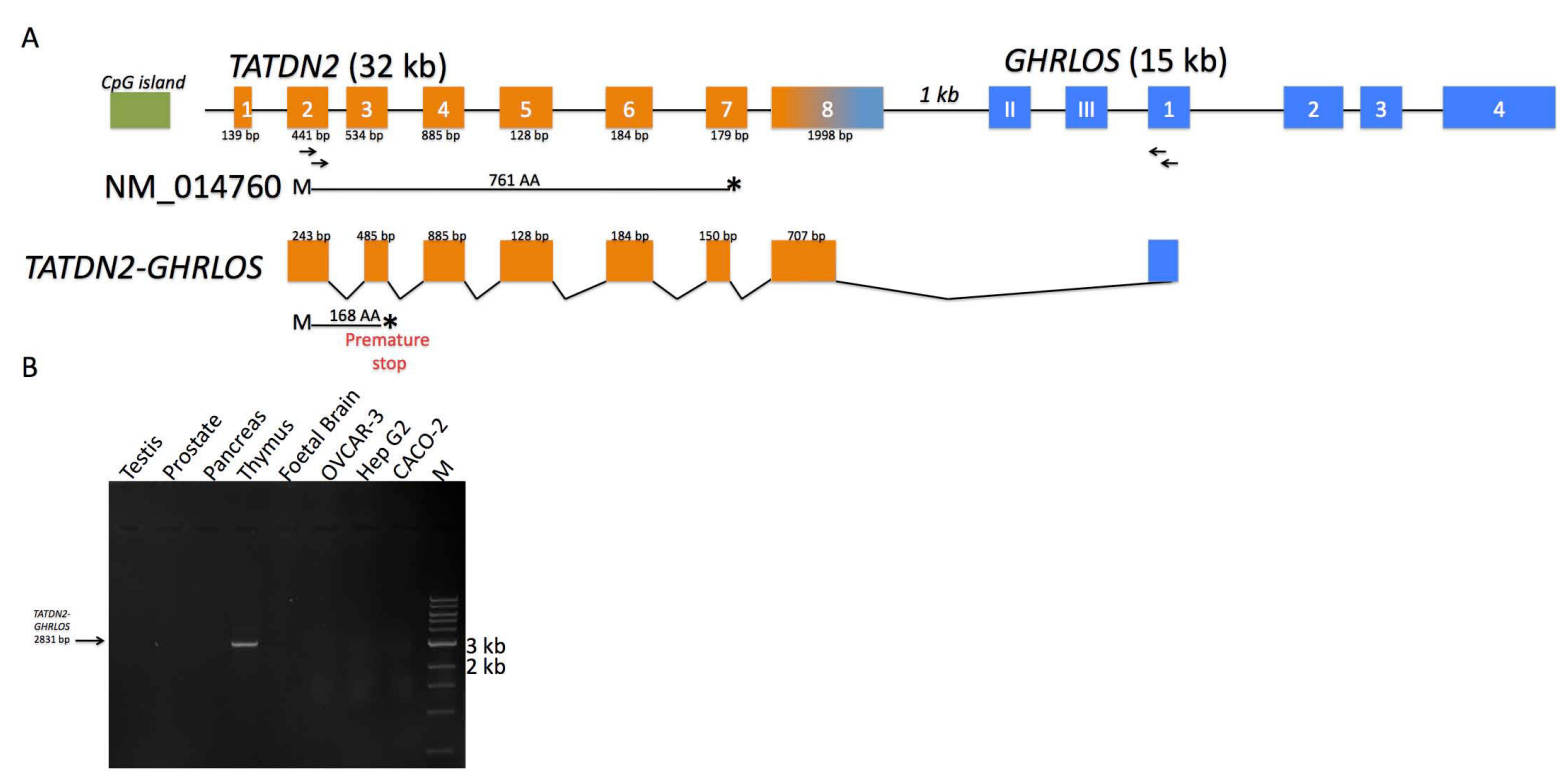
**Identification of a TATDN2-GHRLOS chimaeric transcript by nested RT-PCR**. (A) Overview of the gene structures of *TATDN2 *(orange) and *GHRLOS *exons (blue). *TATDN2 *exon 8 contains first exons of *GHRLOS *and is therefore shown in orange and blue. Exons are represented as boxes, introns as horizontal lines and sizes (bp) are indicated below each exon. A *TATDN2 *CpG island is shown as a green box. The location of RT-PCR primers employed to detect chimaeras are shown below each exon as arrows. The reported 761 amino acid *TATDN2 *open reading frame (encoded by [GenBank:NM_014760]) is shown as a straight line (M = initiating methionine; * = stop codon). A chimaeric *TATDN2-GHRLOS *transcript, its open reading frame and locations of a premature stop codon (*) is shown. Not drawn to scale. (B) Ethidium bromide stained agarose gel of nested RT-PCR product for the *TATDN2-GHRLOS *chimaera. Arrows indicates the amplicon schematically displayed in (A). M = 1 kb DNA Ladder (NEB).

The length of *GHRLOS *transcripts initiating in exon I (present in the 3' UTR of *TATDN2*) is 1.3–3.6 kb, corresponding in size to the Northern data. However, the potential identity of the approximately 5.5 kb transcript seen in the Northern blot with a full-length, 106 nt exon 1 probe (Fig. 2) was not determined. After prolonged exposure of the Northern blot, the 5.5 kb transcript was observed in all tissues except the urinary bladder and uterus (data not shown). *TATDN2 *is only approximately 1 kb upstream of exon II and 4.5 kb from exon 1 of *GHRLOS*, suggesting that transcription-induced chimaeras (TICs) may be generated [20] and give rise to large *GHRLOS *transcripts. Because TICs must contain a first exon of the upstream gene [20], we employed primers in exon 2 (immediately after the start codon) of *TATDN2 *and exon 1 of *GHRLOS *(which was also the sequence of our Northern riboprobe). Using nested RT-PCR (on cDNA reverse transcribed using oligo(dT) primers), we isolated a 2831 bp *TATDN2-GHRLOS *amplicon from the thymus [GenBank:EU789553] (Fig. 4). This transcript has canonical splice donor and acceptor sites (GT/AG) and splices into the expected acceptor site of the 106 bp exon 1. This variant harbour significant open reading frames corresponding to the TATDN2 protein, but contains alternative exons and a premature termination codon more than 50 bp upstream of the final coding exon 7 of *TATDN2 *(Fig. 4). This is likely to result in degradation of the mRNA by nonsense-mediated RNA decay (NMD), a surveillance mechanism that detects and degrades mRNA that may encode truncated proteins with dominant-negative or deleterious gain-of-function activities [21].

We have identified several novel *GHRLOS *transcripts. This includes overlapping *GHRLOS *transcripts initiating in the *TATDN2 *3' UTR and putative transcription-induced chimaeras of *TATDN2 *and *GHRLOS*. These findings extend the previously reported length of *GHRLOS *by ~37 kb. We propose that *GHRLOS *harbour several promoters with start sites in exon 1 (of *GHRLOS*), and in the 3' UTR and the first exon of *TATDN2*.

### Results of in silico analysis indicate that *GHRLOS* is a non-coding RNA gene

**Figure 5:**
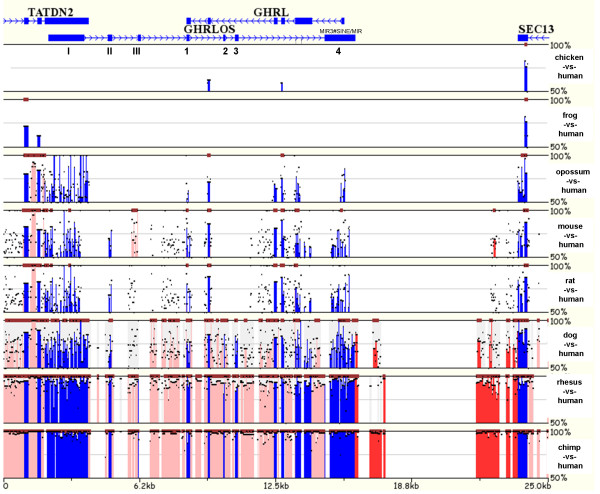
**Mulan sequence conservation profile for vertebrate GHRLOS loci**. The horizontal axis displays the input sequence from human (UCSC hg18), chicken [UCSC galGal3], frog [UCSC xenTro2], opossum [UCSC monDom4], mouse [UCSC mm9], rat [UCSC rn4], dog [UCSC canFam2], rhesus macaque [UCSC rheMac2], and chimpanzee [UCSC PanTro2] *GHRLOS *loci. Evolutionary conserved regions (ECRs, > 70% identity; ≥ 99 bp) are depicted as dark red bars above each pairwise alignment. Exons (blue), intergenic elements (red) and intron sequence (pink) are marked and the vertical axis shows the percent similarity of vertebrate *GHRLOS *orthologues to the human sequence. *GHRLOS *exon numbers are shown. A transposable element in exon 4 (MIR3#SINE/MIR) is shown above exon 4.

Sequence analysis of *GHRLOS *(excluding the putative *TATDN2-GHRLOS *transcription-induced chimaeras that are likely to result in nonsense mediated decay) reveals that *GHRLOS *transcripts do not harbour protein coding potential, but rather have several features of non-coding RNA genes. GMAP analysis showed that *GHRLOS *spans approximately 44 kb on 3p25.3 (data not shown). The Mulan sequence conservation profile for the human and putative vertebrate *GHRLOS *orthologues indicated that most *GHRLOS *exons (I, II, III, 2, 3) are not highly conserved compared to the putative mouse and rat orthologues (Fig. 5). The 106 bp exon 1 (which spans exon 4 of the ghrelin gene) and approximately 90 bp of the 1.4 kb exon 4 (which spans exon -1 of the ghrelin gene) are conserved, however [8]. RT-PCR experiments revealed no evidence for antisense transcription from exon I to exon 1 (of *GHRLOS*) in the mouse (data not shown), suggesting that ghrelin antisense transcription (at these specific locations) may not be conserved. Furthermore, there is very low sequence similarity between the chicken, frog, opossum and human *GHRLOS *sequences (Fig. 5). Interestingly, exons II, 2 and 3 appear to be highly conserved between human and dog [Additional File 5], with 70–75% homology for exons 2 and 3. However, no significant open reading frames (ORFs) are conserved between dog and human *GHRLOS *sequence (data not shown). Taken together, this suggests that *GHRLOS *has evolved rapidly and may be unique to primates.

Our study suggests that *GHRLOS *is a non-coding RNA. *In silico *translation revealed that the heterogeneous *GHRLOS *RNAs contain multiple stop codons, resulting in lack of extensive reading frames, and the putative ORFs do not span conserved regions (data not shown). Moreover, no significant sequence similarity to any known proteins was observed (data not shown). Finally, screening of *GHRLOS *sequence against a reference collection of repeats (RepbasE) using CENSOR [22] identified a 203 bp overlap of exon 4 with the extinct 224 bp MIR3 SINE element, which is present in all vertebrates [23] (Fig. 5). Interestingly, the presence of repeat elements in exons of non-coding RNAs has been reported previously [24-27]

### The *GHRLOS* terminal exon 4 and a putative SEC13 exon overlap in an antisense manner

**Figure 6:**
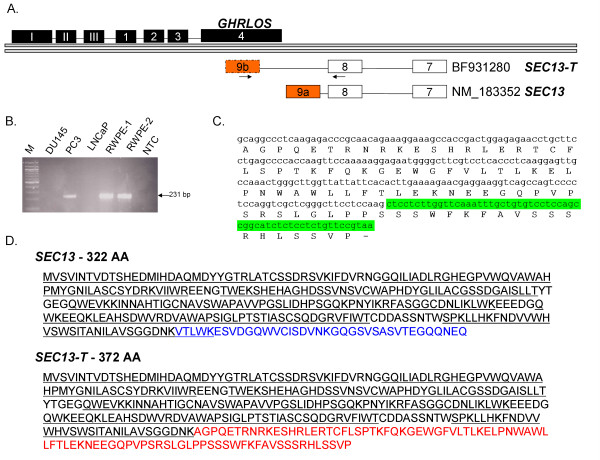
***GHRLOS *overlaps a putative *SEC1*3 isoform, *SEC13-T*, in an antisense manner**. (A) Diagrammatic representation of *GHRLOS, SEC13*, and a novel *SEC13 *isoform, named SEC13-tentative (*SEC13T*). *SEC13-T *contains a novel terminal exon 9, termed exon 9b, which overlaps exon 4 of *GHRLOS *in an antisense manner. In the figure, exon 9b of *SEC13-T *is dotted to indicate that its complete 3' end has not been verified. Exons are represented as boxes and introns as horizontal lines. The locations of the primers employed to verify the *SEC13T *variant are displayed as arrows. *SEC13 *and *SEC13-T *GenBank accession numbers are shown. (B) Ethidium bromide stained agarose gel showing the verification of a *SEC13-T *EST [GenBank:BF931280] transcription by RT-PCR in prostate (RWPE-1 and RWPE-2) and prostate cancer derived cell lines (DU-145, PC3, LNCaP). NTC = no template control (water). M= DNA Molecular Weight Marker VI (Roche). Primer locations are depicted in (A). A 231 PCR fragment was amplified and sequenced, confirming the EST. (C) RNA and amino acid sequence of exon 9b of *SEC13-T*. Additional downstream sequence corresponding to 18 in-frame codons and a stop codon is highlighted in green. (D) SEC13 and SEC13-T amino acid sequences. *SEC13 *encodes a 322 AA protein with six WD-40 repeats (also known as WD or beta-transducin repeats) are underlined. Amino acid sequence of SEC13 which is different to SEC13T is shown in blue. *SEC13-T *may encode a 372 AA protein: Its first 285 amino acids are identical to SEC13 and the normal C-terminal 37 amino acids have been replaced by 87 amino acids (shown in red) encoded by exon 9b.

We have discovered that exon 4 of *GHRLOS *is also on the opposite strand of a novel terminal exon of the neighbouring *SEC13 *gene in a tail-to-tail, 3' to 3', fashion (Fig. 6A) [Additional File 6]. *SEC13 *plays an important role in protein transport and is a component of the COPII complex [28,29]. BLAST analysis identified a brain tumour EST [GenBank:BF931280] [30], which contains exon 7 and 8 of *SEC13*, as well as 206 bp sequence corresponding to a novel exon 9b of *SEC13 *10.7 kb upstream of exon 8. We verified the expression of this EST by RT-PCR (using cDNAs reverse transcribed with oligo(dT) primers) (Fig. 6B) and by sequencing [GenBank:EU789555]. We have named this *SEC13 *variant, SEC13-tentative (*SEC13-T*). *SEC13-T *may encode a 372 amino acid protein. The first 285 amino acids are identical to *SEC13*, while the normal C-terminal 37 amino acids have been replaced by 87 amino acids encoded by the alternative terminal exon 9b (Fig. 6C and 6D). The putative C-terminal-coding exon of the *SEC13-T *isoform appears to be conserved only in primates (data not shown). Therefore *SEC13-T *alternative splicing is likely to be human-specific or primate-specific.

### *GHRLOS* is expressed in many tissues and cell lines and the level of expression shows great variability

**Figure 7:**
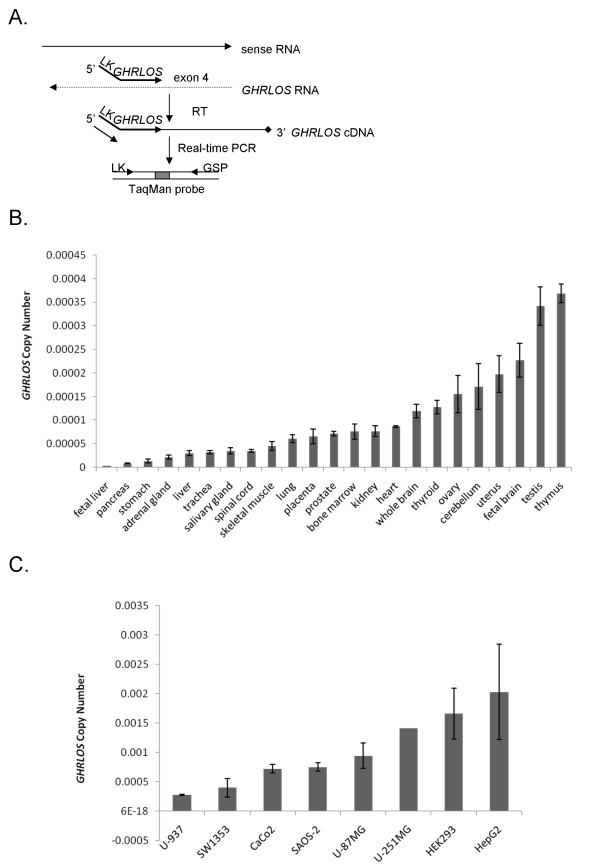
**Strand-specific *GHRLOS *real-time RT-PCR**. (A) Strand-specific real-time RT-PCR approach to quantify *GHRLOS *expression. To select for RNA generated from the antisense strand, a linker sequence (LK) (not present in the genomic sequence of *GHRLOS*) is attached to a *GHRLOS *gene specific primer and employed in reverse transcription. The resulting cDNA is then combined in a real-time PCR, combining a gene-specific primer (GSP) and a primer containing the linker region from reverse transcription primer only. An internal *Taq*Man probe (depicted as a blue box) was employed to increase the specificity and sensitivity of the assay. (B) Relative total *GHRLOS *expression in a range of human human tissues. (C) Relative total *GHRLOS *expression in human cell lines (U-937 non-Hodgkin's lymphoma, SW1353 chondrosarcoma, CaCo-2 colorectal adenocarcinoma, SAOS-2 osteosarcoma, U-87 MG and U-251 MG glioblastoma, HEK293 human embryonic kidney and OVCAR-3 ovarian cancer). Calculations of *GHRLOS *expression levels were performed using the standard curve method (correlating the threshold cycle number (C_T _values) and copy numbers of *GHRLOS*), and normalised to the expression of 18S ribosomal RNA. Each bar presents the mean ± standard deviation of duplicate reactions.

RT-PCR analysis (with primers spanning *GHRLOS *terminal exons) and Northern blotting (which is only suitable for high copy number transcripts) demonstrated that the size of *GHRLOS *transcripts is highly variable, resulting in significant transcript heterogeneity. We, therefore, employed a quantitative, real-time RT-PCR approach in order to more precisely gauge the expression of *GHRLOS *in a range of tissues and cell lines. As the number of alternatively spliced *GHRLOS *transcripts makes it impossible to generate real-time RT-PCR primers that are unique to each splice variant (data not shown), a strand-specific quantitative RT-PCR assay with primers in exon 4 (which is common to all *GHRLOS *variants) was designed to detect total *GHRLOS *RNA expression (Fig. 7A).

The level of total *GHRLOS *transcript expression varied greatly in different human tissues, with high levels in the thymus, testis, foetal brain, uterus, cerebellum, ovary, thyroid, and whole brain. Very low levels of total *GHRLOS *RNA expression were detected in the stomach, foetal liver and pancreas (Fig. 7B). In the thymus the level of expression was approximately 133 fold higher than in the stomach (*P < 0.01*) and 110-fold higher than in the foetal liver (*P < 0.01*). Furthermore, the level of expression in the adult liver was 9-fold higher than in the foetal liver (*P < 0.05*), indicating differential expression according to developmental stage in this tissue. The level of *GHRLOS *in the foetal brain was two-fold higher than the adult brain, but this was not statistically significant (*P > 0.05*).

Expression of *GHRLOS *in a number of continuous cell lines was also examined using real-time RT-PCR (Fig. 7C). Levels of *GHRLOS *expression in the U-937 (Non-Hodgkin's lymphoma) and SW1353 (chondrosarcoma) cell lines were similar to the cerebellum, uterus, foetal brain, testis and thymus. High levels of expression were observed in the HEK293 human embryonic kidney cell line and in the CaCo-2 (colon adenocarcinoma), SAOS-2 (osteosarcoma), U-87 MG and U251-MG (glioblastoma), OVCAR-3 (ovarian adenocarcinoma) and Hep G2 (hepatocarcinoma) cancer cell lines. It is interesting to note that multiple Hep G2 CAGE tags have been identified in the exon I region of *GHRLOS *(see [Additional File 4]). *GHRLOS *RNA appears to be significantly upregulated in the Hep G2 cell line compared to adult and foetal liver (*P < 0.05*) (Fig. 7B and 7C). This observation could indicate that *GHRLOS *may be a specific target in the development of liver and other cancers.

### Comparison of total *GHRLOS* and total *GHRL* expression

**Figure 8:**
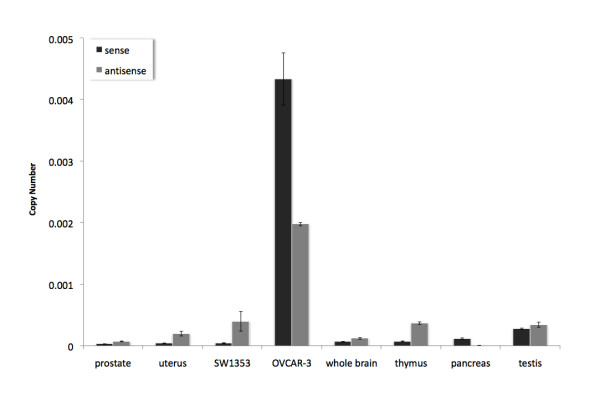
**Comparison of strand-specific *GHRL *and *GHRLOS *real-time RT-PCR**. Relative total *GHRL *(sense, black) and *GHRLOS *(antisense, grey) RNA transcript expression levels in human tissues and cell lines. Calculations of RNA expression levels were performed using the standard curve method (correlating the threshold cycle number (C_T _values) and copy numbers of *GHRL *and *GHRLOS*) normalised to the expression of 18S ribosomal RNA. Each bar presents the mean ± standard deviation of duplicate reactions.

We examined the expression levels of the *GHRL-GHRLOS cis*-natural antisense transcript (*cis*-NAT) pair via quantitative real-time RT-PCR assays detecting total transcription from the ghrelin gene (*GHRL*). As expected [31], the highest level of *GHRL *expression was found in the stomach (data not shown), followed by the testis and pancreas (Fig. 8). When comparing total ghrelin and *GHRLOS *RNA expression in the stomach, *GHRL *was expressed at 2300 fold higher levels than *GHRLOS*, with *GHRLOS *expression almost undetectable (*P < 0.001*). The levels of *GHRLOS *expression were higher than *GHRL *in the thymus, whole brain, the SW1353 chondrosarcoma cell line, uterus and prostate. However, total *GHRL *RNA levels were higher than *GHRLOS *in the pancreas and the OVCAR-3 ovarian cancer cell line.

## Discussion

**Figure 9:**
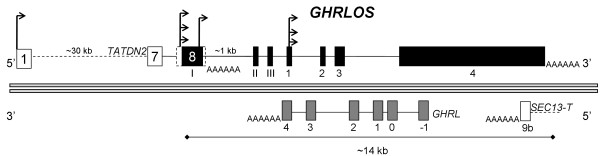
**Structure of genes at the *GHRLOS *locus**. Structure of the human *GHRLOS *(top) and ghrelin (*GHRL*) (bottom) genes. Exons are shown as boxes and introns as lines. Genes on the positive DNA strand are displayed above the double-lines, while genes on the negative DNA strand are shown below. Arrows indicate transcription start sites of GHRLOS transcripts. Ghrelin (*GHRL*) exons are shown in grey, and *GHRLOS *in black. A novel exon of a putative SEC13 variant, SEC13-T is shown as white boxes. The terminal exon 8 of *TATDN2 *is indicated by a dotted line.

Our study demonstrates that *GHRLOS *gives rise to long, extensively spliced, mRNA-like, 5' capped and 3' polyadenylated transcripts suggesting that they are genuine products of RNA polymerase II mediated transcription [32]. We have shown that the *GHRLOS *gene gives rise to transcripts 1.0 to 5.5 kb in size and has many broadly distributed transcription starts sites (TSSs) (Fig. 9). This includes several TSSs in exon 1, TSSs overlapping the 3' UTR of *TATDN2 *and evidence of transcription induced chimaeras employing TSSs in the first exon of *TATDN2*. The ghrelin locus, therefore, gives rise to many antisense transcripts that are currently annotated as a single gene, *GHRLOS*. A well-described example of such complex architecture is the imprinted murine *Gnas *locus, which gives rise to multiple coding and non-coding sense and antisense transcription units [33].

First reported in 1987 [34] and originally thought to be a rarity, it has recently been established that promoters in 3' untranslated regions (3' UTRs) are not uncommon and may be independently transcribed and regulated from their upstream "host gene" [35]. The ENCODE (ENCyclopedia Of DNA Elements) consortium has recently demonstrated that two-thirds of the loci in their dataset contain new putative first exons, which frequently overlap upstream genes [36]. However, it is currently not known how promoters that overlap 3' UTRs are regulated and coordinated [37]. We have demonstrated *GHRLOS *transcription start sites in the 3' UTR of *TATDN2 *via RLM-RACE. The sequence upstream of exon I of *GHRLOS *contains no apparent TATA boxes (data not shown), indicating that *GHRLOS *has a broad type promoter, with many potential transcription start sites in the 2.1 kb 3' UTR of *TATDN2*. This may allow the transcription of numerous tissue-specific and developmental stage-specific transcripts [19]. In addition, multiple CAGE tags are present in the 3' UTR of *TATDN2*, indicating that *GHRLOS *transcripts initiate in this region.

Interestingly, we also report the joining of exons of the neighbouring genes *TATDN2 *and *GHRLOS*. Similar chimaeric transcripts (not caused by chromosomal translocation) have been reported in lower eukaryotes [38,39], but were until recently assumed to be relatively rare in mammals [20,40-43]. It is not known how chimaeric transcripts arise, but transcriptional read-through, followed by canonical *cis*-splicing is the most likely mechanism [40]. Alternatively, chimaeric transcripts could arise through *trans-*splicing, but the existence of this mechanism has not been well-established in mammals [44]. We, therefore, suggest that the chimaeric *TATDN2-GHRLOS *transcripts probably result from a single pre-mRNA, creating transcription-induced chimaeras (TICs) which are spliced in *cis *[40]. The *TATDN2-GHRLOS *TIC which was identified in the normal thymus harbours premature stop codons (PTCs) and this is likely to result in nonsense-mediated decay (NMD). NMD is an RNA surveillance mechanism (present in eukaryotes) which targets mRNA with premature stop codons that, if translated, encode truncated proteins with dominant-negative or deleterious gain-of function activities [21]. Whilst we are not aware of any studies of the translated product of *TATDN2 *(TatD DNase domain containing 2), TatD is a cytoplasmic protein that harbours magnesium-dependent DNase activity in *E. coli *[45]. Proteins with a TatD domain, therefore, belong to the large superfamily of metalloenzymes. This suggests that the chimaeric transcripts may play a role in auto-regulation of the *TATDN2 *protein product, which we hypothesise is likely to be a DNase associated with apoptosis. We speculate that *TATDN2-GHRLOS *chimaeras may be used to tightly regulate the programmed cell death of T-cells by downregulating the translation of the putative DNase TATDN2. Hillman and colleagues have termed this system of auto-regulation, where alternative splicing results in mRNA isoforms that are not translated, regulated unproductive splicing and translation (RUST) [46]. Interestingly, it has very recently been reported that transcripts of the tumour suppressor protein E-cadherin that result in non-sense mediated decay are upregulated in gastric cancer, suggesting that NMD may also promote disease [47].

We previously reported that *GHRLOS *completely overlaps the ghrelin (*GHRL*) gene [8]. Here we also show that the 3' terminal exon 4 of *GHRLOS *is present on the opposite (antisense) strand to a novel, 3' terminal *SEC13 *exon (Fig. 8). SEC13 is a protein that forms a part of the coat protein complex II (COPII) [28]. COPII proteins are required for the trafficking of nascent proteins from the endoplasmic reticulum (ER) to the Golgi apparatus. It also plays a role in the selection and concentration of cargo proteins for transport [29]. SEC13 [28], therefore, has a core endocrine function. *GHRLOS *overlaps a novel *SEC13 *variant, SEC13-tentative (*SEC13-T*). *SEC13-T *may encode a 372 amino acid protein with a unique C-terminus. Future studies are required to confirm the presence of full-length transcripts and to elucidate the functional role of SEC13-T protein. We suggest that transcription of *SEC13-T *may have implications for COPII function in health and disease states. As *GHRLOS *overlaps the terminal exons of both *GHRL *and *SEC13-T*, the *GHRLOS *natural antisense transcripts may regulate one or both of these genes.

### *GHRLOS*, a candidate non-coding RNA

Non-coding RNAs are frequently not conserved between species, suggesting that they are either biological noise (non-functional transcription), or that they have species specific-functions. Species-specific non-coding transcripts have been observed and there is strong evidence that non-coding transcripts are functionally significant [48-52]. Interestingly it has recently been observed, using *in silico *analysis, that even between closely related *Drosophila *species non-coding RNAs are not conserved [53]. While the number of protein coding genes in distant eukaryotes (such as worms, mice, and humans) is approximately equal, the relative amount of non-coding DNA increases in proportion to eukaryotic complexity [54,55]. Mattick and colleagues explain this paradox by hypothesising that non-coding RNAs have evolved to enable the emergence of organisms with increasingly complex higher levels functions [55,56]. Our *in silico *analysis suggests that *GHRLOS *exons show very low sequence conservation in vertebrate species. Furthermore, exon 4 of *GHRLOS *contains a transposable element, a feature observed in many non-coding RNAs [25-27].

Our bioinformatic studies indicate that *GHRLOS *does not encode a protein and, therefore, is a non-coding RNA. Taking into account the full-length sequence of *GHRLOS*, the length of the open reading frames is very short. Moreover, *GHRLOS *RNAs are numerous (due to extensive splicing), contain a large number of stop codons and there is little nucleotide and putative amino acid conservation between vertebrates, making it less likely that this gene encodes proteins. Although it has been suggested that many small peptides may be translated [57], the majority of small peptides are processed from larger precursor proteins, as is ghrelin itself which is processed from preproghrelin. Therefore, while it cannot be excluded that *GHRLOS *encodes short ORFs (that are not conserved in the mouse) it appears unlikely that *GHRLOS *encodes biologically active peptides.

We examined the *GHRLOS *expression profile to strengthen the hypothesis that it is a candidate non-coding RNA. It has been demonstrated that mammalian long non-coding RNAs are expressed in a tissue-specific manner, indicating that they are biologically significant [50,58-61]. In both humans and mice, the major tissues of non-coding expression are the complex organs; the brain, testis, and thymus [50,61]. This also holds true in *Drosophila*, where the majority of the candidate non-coding RNAs are expressed in the central nervous system [53]. Indeed, non-coding RNAs are emerging as important regulators of complex systems, such as the central nervous system (brain) and intricate processes, including spermatogenesis in the testis [62-65]. We demonstrated high levels of *GHRLOS *in the thymus, brain, testis, uterus, ovary and thymus, while the expression levels in the stomach, where *GHRL *is highly expressed [1], were almost undetectable. Our data demonstrate that *GHRLOS *is predominantly expressed in a limited number of tissues and cell types, suggesting that these transcripts have physiological functions in distinct cell types and tissues. Moreover, real-time RT-PCR showed extremely low levels of *GHRLOS *in the foetal and adult liver and high levels in the Hep G2 hepatocarcinoma cell line, suggesting that *GHRLOS *expression may be altered in liver cancer. Indeed, it has recently been reported that non-coding RNA expression is frequently altered in cancer [58,66]. This indicates that non-coding RNAs may have specific functions in normal cells. Much like protein-coding transcripts, ncRNAs may act as tumour suppressors, or be upregulated in cancer and act as oncogenes. An examination of *GHRLOS *expression in cancer would, therefore, be of great interest.

Here we have characterised the structure and organisation of *GHRLOS*, a ghrelin antisense gene, suggesting that *GHRLOS *has multiple first exons. Therefore, it is possible that *GHRLOS *could be a part of very large, continuous ncRNA species in the 3p25 chromosomal region and beyond. In the absence of hallmarks, such as large open reading frames, mapping complex non-coding RNA genes remains a complex task. For example, two RNAs in the *FMR1 *locus, the *cis*-NAT *ASFMR1 *[67] and the non-coding RNA gene *FMR4 *(found just upstream of *FMR1 *[51]) may be one continuous RNA isoform [51].

### What is the function of *GHRLOS*?

It is currently difficult and costly to determine the mechanism of function of long non-coding RNAs. The roles of non-coding RNAs are likely to be diverse, and there is strong evidence that they play a role in regulating important pathways. Many ncRNAs are expressed during development, neural differentiation, during macrophage activation and in cancer, indicating that they have key functions in these processes [12,63,68]. Non-coding RNAs have been found to play a role in the silencing of overlapping genes in *cis *[69], in the silencing of distant chromosome regions in *trans *[70], in nuclear trafficking [71], apoptosis [51,72], promoter repression [73], and can act as tumour suppressors [74]. Moreover, ncRNAs are emerging as markers for complex human disease, including lung cancer [75], heart disease [76], and a range of other pathologies [68,77]. *GHRLOS *may also serve as a host gene for snoRNA (small nucleolar RNAs) genes [78] or *GHRLOS *RNA transcripts may be precursors for short RNAs, such as micoRNAs [77], endogenous siRNAs [79,80], piRNAs [81] and other novel, short non-coding RNA species [82,83].

Although the understanding of natural antisense transcripts (NATs) remains in its infancy, they have been associated with a range of regulatory mechanisms that are not necessarily mutually exclusive. This includes transcriptional interference, RNA masking and dsRNA mediated gene-silencing via direct interaction between the sense and antisense transcripts [9,84,85].

While it is difficult to predict *GHRLOS *function, the fact that all spliced *GHRLOS *variants share exon 1, which overlaps the 3' untranslated exon 4 of *GHRL *is striking. Our findings suggest that *GHRLOS *functions as a non-coding RNA. There are a few examples that suggest that antisense transcripts are important in the regulation of endocrine hormone receptors, including a thyroid hormone receptor [86,87], and the luteinising hormone/choriogonadotropin receptor [88] gene and in the regulation of growth factors [89]. Interestingly, it has been recently suggested that the invertebrate (insect) polypeptide hormone allatostatin may be regulated by *cis*-NATs [90]. To date, however the physiological and pathophysiological roles of natural sense/antisense pairs have not been elucidated for any vertebrate endocrine hormone. Further studies are necessary to reveal the function and molecular mechanisms regulating the candidate non-coding RNA gene *GHRLOS*.

## Conclusion

In the present study, we have characterised *GHRLOS*, which gives rise to endogenous ghrelin natural antisense transcripts. *GHRLOS *exhibits features which are common to many non-coding RNA genes, including extensive splicing, lack of significant and conserved open reading frames, differential expression and lack of conservation in vertebrates. Our data also reveal that *GHRLOS *contains multiple first exons and that it overlaps both *GHRL *and a novel *SEC13 *exon in the antisense direction, suggesting that *GHRLOS *may have a role in regulating these genes. Moreover, we report *TATDN2-GHRLOS *chimaeras that may function to regulate the translation of the putative DNase TATDN2. Additional studies are underway to elucidate the functions of *GHRLOS *and to investigate, in particular, its overlapping genomic arrangement with the ghrelin gene. These studies may provide a new, physiologically relevant model system for investigating the roles of antisense gene and non-coding RNA regulation and the mechanisms involved, as well as establishing whether *GHRLOS *RNAs may be useful markers for diagnosis and prognosis of complex disease.

## Methods

### Bioinformatics

Multiple sequence alignments were generated using the MUltiple sequence Local AligNment and conservation visualization tool (Mulan) [91]. Human [UCSC hg18], chicken [UCSC galGal3], frog [UCSC xenTro2], opossum [UCSC monDom4], mouse [UCSC mm9], rat [UCSC rn4], dog [UCSC canFam2], rhesus macaque [UCSC rheMac2], and chimpanzee [UCSC PanTro2] genomic sequences were obtained via the Evolutionary Conserved Regions (ECRs) Browser [92] and forwarded to Mulan to generate a full local alignment of the *GHRLOS *locus. *GHRLOS *was annotated based on the exons sequenced in this study.

To identify putative antisense exons and transcripts, we examined the publicly available Affymetrix Human Exon 1.0 ST Array tissue panel dataset consisting of 11 tissues (breast, cerebellum, heart, kidney, liver, muscle, pancreas, prostate, spleen, testes, and thyroid) using the Affymetrix All Exon track in the UCSC Genome Browser [93] and the Integrated Genome Browser (IGB) from Affymetrix [94].

To locate transcription start sites in the putative first exons of *GHRLOS*, CAGE (Cap Analysis of Gene Expression) tags (deposited by the RIKEN consortium and its collaborators) were obtained via the Genome Network Platform Viewer [95]. We then recovered the RNA library information for each CAGE tag starting site. Briefly, each CAGE tag was individually queried against the 1.4 GB CAGE tag sequencing file, (release date 13.11.2006) available on the Genome Network Platform website using the UNIX grep command [96].

The exon-intron-structure of ESTs and mRNA entries identified from BLAST searches, as well as sequenced PCR amplicons obtained in this study, were analysed against the human genome (NCBI release 35) using GMAP [14]. Presence of open reading frames was analysed by NCBI ORF Finder [97], Fickett's TestCode [98,99] and ESTScan2 [100,101]. The presence of transposable elements in *GHRLOS *sequence was examined using CENSOR v4.2.8 [22]. Protein domain analysis was performed using the SMART database [102].

### Cell culture and RNA extraction

The following cell lines (originally obtained from the American Type Culture Collection/ATCC, Rockville, MD unless specified) were cultured in their recommended media: Prostate and/or prostate cancer derived cell lines DU145 (ATCC HTB-81), RWPE-1 (ATCC CRL-11609), RWPE-2 (ATCC CRL-11610), LNCaP (ATCC CRL-1740), 22Rv1 (ATCC CRL-2505) and PC3 (ATCC CRL-1435), HEK293 human embryonic kidney (ATCC CRL-1573), SAOS-2 osteosarcoma (ATCC HTB-85), Hep G2 hepatocarcinoma (ATCC HB-8065), U-87 MG and U-251 MG glioblastoma (ATCC HTB-14 and JCRB Cell Bank # IFO50288, respectively), CaCo-2 colorectal adenocarcinoma (ATCC HTB-37), SW1353 chondrosarcoma (ATCC HTB-94), and OVCAR-3 ovarian cancer (ATCC HTB-161). All cells were grown in T80 or T175 flasks (Nagle Nunc International, Roskilde, Denmark) in 95% CO_2 _in a Sanyo incubator at 37°C. Total RNA was harvested from cultured cells at 70% confluence using TRIzol reagent (Invitrogen, Carlsbad, CA) according to the manufacturer's instructions.

### Human RNA samples

Tissue Total RNA was obtained from the stomach, prostate (FirstChoice, Ambion, Austin, TX), foetal liver, adrenal gland, liver, trachea, salivary gland, spinal cord, skeletal muscle, lung, placenta, bone marrow, kidney, heart, whole brain, thyroid, cerebellum, uterus, foetal brain, testis, thymus (Human total RNA Master Panel II, Clontech, Mountain View, CA) and pancreas (Clontech).

### 5' and 3' RACE mapping of *GHRLOS *transcripts

To further characterise the 5' end of the putative ghrelin antisense RNAs, 5' RACE was undertaken using FirstChoice RLM-RACE-Ready human stomach cDNA (Ambion) according to the manufacturer's instructions. The first round PCR was performed with an adapter-specific sense primer (5'adapter-out-F, Table 1) and an exon 2-specific antisense primer (5'OS-out-R in Table 1). PCR product (1 μl) was used in a secondary, nested PCR with a gene specific primer in exon 2 (5'adapter-in-F and 5'OS-in-R, Table 1). PCRs were performed in a total reaction volume of 50 μl using 1 U of Platinum *Taq *Polymerase High Fidelity (Invitrogen) according to the manufacturer's instructions.

**Table 1:** Designations and sequences of oligonucleotides

Name	Sequence (5'-3')	GHRLOS Exon	Ta (°C)	PCR Cycles
5'2-out-F	GATGGCGATGAATGAACACTG	N/A		
5'2-out-R	AATCATCTCAGGAATACCTGGA	2	60	35
5'2-in-F	ATGAATGAACACTGCGTTTGC	N/A		
5'2-in-R	AAATGGAAGAGATGAGGCGC	2	61	35
Ex1-cRNA-F	CATACAGTTTGAACATTTATTCGCCTCC	1		
Ex1-cRNA-R	CTAATACGACTCACTATAGGGAGACTCTCTCTAAGTTTAGAAGCGCTCATCTG	1	62	25
3'2OF	GAGAGCGCCTCATCTCTTCC	2		
3'2OR	GCGAGCACAGAATTAATACGACTC	N/A	63	35
3'2IF	ATGATTTATTGGAGCTCAAAGC	2		
3'2IR	GAATTAATACGACTCACTATAGGT	N/A	57	15
3'4	TACGGAACAGAGGAGAGATGC	4	60	35
3' -RACE-adapter	GCGAGCACAGAATTAATACGACTCACTATAGGTTTTTTTTTTTTVN	N/A		N/A
IPCR-out-F	AAATCCCACCTTTAGTCCCA	4	60	35
IPCR-in-F	CTGCCACCTGAGTGTAGAC	4	60	20
IPCR-ALL-R	CACAGGCTTGGAGACTTCC	I		
Pthioate-hex	NNNNsNsN	N/A	30	N/A
Phospho-dT	P- GGCCACGCGTCGACTAGTAC(T)_18_	N/A	55	N/A
GHRL-Real-RT-LK	CGACTGGAGCACGAGGACACTGAGCCAGAGAGCGCTTCTAAACTTA	N/A	N/A	
GHRL-Real-F	GCCCCAGCCGACAAGTG	N/A	60	40
Ito4-F	CATGGAAGTCTCCAAGCCTG	I		
Ito4-R	CTGCTCTACTGCCTCAATGTC	4	63	34
GHRLOS-Real-RT-LK	CGACTGGAGCACGAGGACACTGACAATCCTCCCTGAGGTTGATCT	4	N/A	
GHRLOS-Real-F	CATTGAGGCAGTAGAGCAGTTGA	4		
LK	CGACTGGAGCACGAGGACACTGA	N/A	60	40
Ex4-TaqMan	FAM-TGCCGAATGACCACCTACCCTGACTT- BHQ1	4		
18S-Real-F	TTCGGAACTGAGGCCATGAT	N/A		
18S-Real-R	CGAACCTCCGACTTTCGTTCT	N/A	60	40
F_CAGE	GGGACTGCCTGTAATAGCAC	I		
R_CAGEout	CACGACTGTTGTACAAGCTC	1	60	35
R_CAGEin	GGAGGCGAATAAATGTTCAAACTG	1	61	30
ChiOut-F	TGAAAGCCCAGAAGGAGGA	N/A		
ChiOut-R	TCTAAGTTTAGAAGCGCTCATCTG	1	63	35
ChiIn-F	CAGAAGGAGGACGATGTGG	N/A		
ChiIn-R	CACGACTGTTGTACAAGCTC	1	62	30
UIS231-F	ACAAGTTCAACGATGTGGTG	N/A		
UIS231-R	CAAGTGTGAATAATAACCAAGCCC	N/A	55	40

T7 promoter sequence in PCR primers is underlined. Linker sequence (LK) in the primer GHRLOS-Real-RT-LK and GHRL-Real-RT-LK is shown in bold. In primer 3' -RACE-adapter V denotes an A/G/C residue and N denotes A/G/C. Lowercase letters in primer Pthioate-hex denotes phosphothioate linkages. An uppercase P in primer Phospho-dT indicates that the synthetic oligonucleotide was 5' phosphorylated. Annealing temperatures (Ta) of oligonucleotides employed in PCR is shown. The *GHRL *or *GHRLOS *exon location of nucleotides are listed, while oligonucleotides spanning synthetic sequences (adapters and linkers) or genes other than *GHRL *and *GHRLOS *are denoted as N/A.

For *GHRLOS *3' RACE, human stomach and PC3 prostate cancer cell line total RNA was reverse transcribed using Transcriptor reverse transcriptase (Roche Applied Science Applied Science, Penzberg, Germany) and 10 μM adapter primer (3'-RACE-adapter, Table 1) from the FirstChoice RLM-RACE Kit (Ambion). 3' RACE was performed with 2 μl of this cDNA. Two 3' RACE reactions were performed – one combined an exon 4 *GHRLOS*-specific forward primer and an adapter-specific reverse primer (3'4F and 3'2OR, Table 1), and the other used an adapter-specific reverse primer and an exon 2-specific forward primer 3'2OR/F, Table 1). PCR products were then diluted and used in a secondary, nested PCR with a gene-specific forward and a reverse adapter primer (3'2IF/R, Table 1). PCR products were purified using a High Pure PCR purification kit (Roche Applied Science), cloned into *pCR-XL-TOPO *(Invitrogen) or *pGEM-T Easy *(Promega, Madison, WI), transformed into DH5α chemical competent cells (Invitrogen) and sequenced at the Australian Genome Research Facility (AGRF, Brisbane, Australia) using BigDye III (Applied Biosystems, AB, Foster City, CA).

### Determination of *GHRLOS *transcription start and polyadenylation sites by Rolling Circle Amplification Rapid Amplification of cDNA Ends (RCA-RACE)

To simultaneously obtain the 5' and 3' ends of *GHRLOS *transcripts, we employed Rolling Circle Amplification-RACE (Rapid Amplification of cDNA Ends [103], an improved inverse PCR approach. Briefly, 3 μg stomach, prostate, RWPE-1 cell line and PC3 prostate cancer cell line total RNA were reverse transcribed using 10 U of Transcriptor reverse transcriptase (Roche Applied Science) and 100 μM HPLC-purified 5'-end phosphorylated oligo d(T)-adapter primer (Phospo-dT, Table 1) (Proligo, Boulder, CO) according to the manufacturer's instructions. The single-stranded cDNA was purified using a High Pure PCR purification kit (Roche Applied Science) and eluted in 50 μl elution buffer (10 mM Tris-HCl, pH 8.5). Next, 25 μl purified linear cDNA was circularised using 100 U of CircLigase (EPICENTRE Biotechnologies, Madison, WI) and purified as before. After self-ligation, 15 μl circular cDNA was added to a rolling circle amplification reaction with 10 U of φ 29 DNA polymerase (NEB) and 10 μM HPLC-purified random hexamer primers with two phosphothioate linkages on their 3'ends (Pthioate-hex, Table 1) (Proligo). Following a 21 h incubation at 30°C in a waterbath, RCA-products were subjected to two rounds of inverse PCR with 1 U of Platinum *Taq *HIFI polymerase (Invitrogen), as per manufacturer's instructions. PCRs were performed in a reaction volume of 50 μl using a PTC-200 thermocycler (MJ Research, Waltham, MA). For the first round amplification, the RCA reaction was diluted 1/100 in water, and 1 μl used in an outer PCR with a forward primer at the start of exon 4 and a reverse primer in exon I (IPCR-out-F and IPCR-ALL-R, Table 1). After 35 cycles at a 60°C annealing temperature, the outer PCR product was diluted 100 times in water and 1 μl was used in a hemi-nested PCR of 20 cycles, with annealing at 60°C (IPCR-in-F and IPCR-ALL-R, Table 1). Amplification products were eluted from agarose gels in 50 μl water overnight, reamplified, cloned into *pCR-XL-TOPO *(Invitrogen), transformed into One Shot MAX Efficiency DH5α-T1R chemically competent cells (Invitrogen) and sequenced at the Australian Genome Research Facility (AGRF, Brisbane, Australia).

### Northern blot hybridisation

Initially, a cRNA probe spanning exon I, II, 1 and 2 of *GHRLOS *was employed. Briefly, a 5' RACE clone in pGEM-T Easy was linearised with *SalI *restriction enzyme and a cRNA probe was synthesised using T7 RNA polymerase and a digoxigenin (DIG) RNA labelling kit (Roche Applied Science). Probe concentration was estimated by dot blot comparison with digoxigenin-labelled standards. 500 ng stomach poly(A)^+ ^RNA (FirstChoice, Ambion) was separated on a 1.2% formaldehyde gel and blotted, as described previously [103]. Samples were electrophoresed with 50 ng RNA Molecular Weight Marker II (Roche Applied Science). The blot was hybridised to 50 ng/mL DIG-labelled cRNA probe overnight. Prehybridisation and hybridisation was performed with DIG-Easy Hyb (Roche Applied Science) at 65°C. The membranes were washed twice for 5 min at room temperature with 1 × Saline-Sodium Citrate (SSC), 0.1% sodium dodecyl sulfate (SDS) and then washed three times for 10 min at 65°C with 0.1 × SSC, 0.1% SDS. The membrane was then reacted with an alkaline phosphatase (AP)-conjugated anti-DIG antibody (Roche Applied Science). AP activity was detected using a chemiluminescence method using CDP-Star (Roche Applied Science).

A second cRNA probe, which spanned exon 1 (which is common to all known *GHRLOS *mRNA isoforms) was synthesised from 100 ng human stomach genomic DNA (BioChain, Hayward, CA) using the PCR method [104] (Ex1-cRNA-F/R, Table 1). The PCR product was purified using a High Pure PCR Product Purification Kit (Roche Applied Science) and the DIG-labelled cRNA probe synthesised and quantified as detailed above. A multi-tissue membrane containing poly(A)^+ ^RNA from 12 human tissues (brain, duodenum, oesophagus, pancreas, PBL/leukocytes, prostate, salivary gland, testis, thymus, thyroid, urinary bladder and uterus) was purchased from OriGene (Rockville, MD). Prehybridisation and hybridisation were performed as described above, except that ULTRAhyb Ultrasensitive Hybridization Buffer (Ambion) was used instead of DIG-Easy Hyb (Roche Applied Science). Equivalent loading between tissues on the blot was determined by rehybridising with 20 ng/mL DIG-labelled β-actin cRNA probe (Roche Applied Science).

### Isolation of alternatively spliced *GHRLOS *mRNAs via non-quantitative RT-PCR

For non-quantitative RT-PCR analysis of *GHRLOS *splicing, RT-PCRs were performed with a forward primer in a region common to the 5' terminal exon Ia/b and a reverse primer in the 3' terminal exon 4 of *GHRLOS *(Ito4-F/R, Table 1). cDNA was synthesised in a final volume of 20 μl from 3 μg total RNA from tissues and cell lines using 10 U of Transcriptor reverse transcriptase (Roche Applied Science), 20 U of RNasin Plus RNase Inhibitor (Promega) and a 3' RACE adapter primer (3'-RACE-adapter, Table 1) at 55°C according to the manufacturer's instructions. PCR amplicons from the stomach, prostate, foetal brain, heart, thymus, testis, and pancreas were purified, sub-cloned and sequenced as described above.

### Long-range RT-PCR to detect putative chimaeric *TATDN2-GHRLOS *transcripts

To detect long, chimaeric transcripts, we employed RT-PCR with a forward primer in exon 2 of *TATDN2 *(ChiOut-F, Table 1) and a reverse primer in exon 1 of *GHRLOS *(ChiOut-R, Table 1). PCR was carried out with 1 U of Platinum *Taq *HIFI polymerase (Invitrogen) as per manufacturer's instructions, extending at 68°C for 2.5 minutes per cycle. cDNA was synthesised as above in a final volume of 20 μl from 2 μg total RNA, from the Hep G2 hepatocarcinoma cell line, CaCo-2 colorectal adenocarcinoma cell line, OVCAR-3 ovarian cancer cell line, and from a range of normal tissues (testis, prostate, pancreas, thymus, and foetal brain). RT-PCR products were sub-cloned and sequenced as described above.

### CAGE-aided cDNA primer walking

To determine if the identified upstream CAGE tag starting sites transcribe exons that belong to *GHRLOS*, we employed RT-PCR using a forward primer designed to the region immediately after a CAGE cluster in the ~2 kb 3' untranslated region of the adjacent gene *TATDN2 *(TSS ID T03F009D1927) and a reverse primer in exon 1, an exon which is common to all known *GHRLOS *variants (F_CAGE and R_CAGEout in Table 1, respectively). cDNA was synthesised in a final volume of 20 μl from 2 μg total RNA, from the Hep G2 hepatocarcinoma cell line and the thymus and foetal brain, using 10 units Transcriptor reverse transcriptase (Roche Applied Science), 20 U of RNasin Plus RNase Inhibitor (Promega) and oligo(dT)_18 _primers (Proligo) according to the manufacturer's instructions. The cDNAs were subjected to 35 cycles of PCR with a two-minute extension time per cycle, and then diluted 1/100 in water and subjected to a hemi-nested 30-cycle PCR with a nested primer in exon 1 (R_CAGEin, Table 1). PCRs were performed in a total reaction volume of 50 μl using 10 U of Platinum *Taq *Polymerase High Fidelity (Invitrogen) according to the manufacturer's instructions. Entire PCR products were purified using the High Pure PCR Product Purification Kit (Roche Applied Science), subcloned into *pCR-XL-TOPO *(Invitrogen) and transformed into One Shot MAX Efficiency DH5α-T1R chemical competent cells (Invitrogen). Insert-positive, purified clones were sequenced by the Australian Genome Research Facility (AGRF, Brisbane, Australia) using the AB PRISM BigDye Terminator Cycle Sequencing Kit v3.1 protocol (AB).

### Identification of novel *SEC13 *exon

To verify the presence of a novel *SEC13 *exon identified in a brain tumour EST [GenBank:BF931280], cDNAs reverse transcribed with an oligo(dT) primer (as described above) were challenged by RT-PCR with primers in exon 8 of *SEC13 *and a reverse primer in the novel exon (231-F/R, Table 1, respectively).

### Strand-specific, quantitative real-time RT-PCR

To allow strand-specific and RNA-specific amplification [105-107] of *GHRLOS *transcripts, reverse transcription was performed using a gene-specific primer in exon 4 with a linker (LK) [107] sequence attached to the 5' end of the primer (*GHRLOS*-Real-RT-LK, Table 1). cDNA was generated from 1 μg total RNA using 40 U of AMV reverse transcriptase (Roche Applied Science) at 42°C, according to the manufacturer's instructions. The strand-specific, real-time RT-PCR was performed with an exon 4 specific forward primer, a reverse primer with the LK sequence only (*GHRLOS*-Real-F and LK, Table 1) and a TaqMan probe (Ex4-TaqMan, Table 1). To detect sense *GHRL *transcripts, we employed a strand-specific RT-PCR approach, with a reverse transcription primer spanning the 3' terminal exon 4 of the ghrelin gene (GHRLex4_RT_LK, Table 1) followed by PCR with an exon 4 specific forward primer (GHRLex4_F, Table 1) and a linker-specific reverse primer. (LK, Table 1). The relative quantification of *GHRLOS *and *GHRL *transcripts was estimated by direct normalisation to the threshold cycle (C_T_) of the housekeeping gene, 18S ribosomal RNA (18S-Real-F/R, Table 1). *18S *PCRs were used to normalise real-time data. As reported for *GAPDH *[107], *18S *RNAs self-primes efficiently in reverse transcription reactions without the addition of random or gene-specific primers. All primers were designed using the Primer Express version 2.0 software (AB).

PCRs were performed in a total reaction volume of 20 μl using Platinum Quantitative PCR SuperMix-UDG w/ROX (Invitrogen) for *GHRLOS*, while *GHRL *and the housekeeping gene 18S ribosomal RNA were amplified using 2 × SYBR green master mix (AB). Controls included the use of cDNA, which was reverse transcribed using random hexamers as primers, as well as the reverse transcription of RNA in the absence of primer. Real-time RT-PCR was performed using the AB 7000 sequence detection system (AB) and data analysed using the absolute standard curve method (User Bulletin #2, AB) to determine expression levels in a range of tissues and cell lines. Briefly, we calculated values from duplicate reactions for each sample from standards, which were constructed from PCR products. Statistical significance was determined using the Student's t-test and, where applicable, one-way analysis of variance (ANOVA) with Tukey post-hoc analysis. P-values of < 0.05 were considered to be statistically significant. Data are represented as mean ± standard deviation (S.D.).

## Authors' contributions

IS conceived and designed the study and carried out all experiments except quantitative real-time RT-PCR. SLC carried out real-time RT-PCR and the resulting statistical analyses. ACH and LKC participated in its design and coordination. All authors participated in interpreting the data, wrote, read and approved the final manuscript).

## Supplementary Material

Additional file 1 Overview of *GHRLOS *exon 1. This is a TIFF file showing *GHRLOS *transcription start sites and exon 1 sequence. Exon 1 and exons I, II and III, all of which splice into the 106 bp exon 1, are depicted as black boxes. Exons with transcription start sites are indicated by arrows in the direction of transcription. The sequence of the 106 bp exon 1, which splices into upstream exons, is shaded in grey. Transcription start sites in exon 1, previously determined via 5' RACE (Δ) and CAGE (Cap Analysis of Gene Expression) (*), are indicated, and the exact transcription start site nucleotides are underlined and in bold. For comparison, the sequence of the reference (sense) exon 4 of the ghrelin gene [GenBank:NM_016362] is shown in red. *GHRLOS *exon 1 sequence is shown in upper case, while intron sequence is shown in lower case. Click here for file

Additional file 2 Ethidium bromide stained agarose gel electrophoresis of *GHRLOS *exon I a/b to 4 non-quantitative RT-PCR amplicons from cultured cells and normal prostate and stomach tissue. This is a TIFF file showing the expression profile of exon I a/b to 4 amplicons in various human tissues and cell lines, indicating a complex splice pattern. M = MassRuler Express DNA ladder (Fermentas, Burlington, Canada). Click here for file

Additional file 3 Compilation of exons of *GHRLOS *transcripts. This is a PDF file listing exons of *GHRLOS*. Exon and intron sizes (bp) are indicated. Experimental evidence from this study and/or external references for each exon are shown. Click here for file

Additional file 4 Overview of CAGE-aided primer walking amplicons and CAGE tags in the 3' untranslated exon 8 of *TATDN2*. This is a PDF file showing (A) The expression pattern of transcripts spanning exon 8 of *TATDN2 *(orange) and exon 1 of *GHRLOS *(blue) revealed via cDNA primer walking (exon shown as boxes, introns as lines, and sizes in bp indicated above each exon). (B) CAGE tags present in the 3' untranslated region of *TATDN2 *(H51BA34H0302-testis; H22BA47L0809-Hep G2 hepatocarcinoma; H21BB07A0112-Hep G2; H52BA49L1810-adrenal gland; H04BB25B1106-cerebrum; H63BA79F2401-heart; H59BA94L1109-SK-N-AS neuroblastoma cell line; H08BA37K2001-kidney malignancy; H21BA90I0206-HepG2 hepatocellular liver carcinoma; H62BB07A0901-SK-N-AS). *TATDN2 *exon sequence is shown in beige, intron sequence in lowercase and grey, *GHRLOS *sequence deduced from cDNA primer walking in orange, and the *TATDN2 *polyadenylation signal (AATAAA) is shown in red. Click here for file

Additional file 5 Comparison of human (h) and putative dog *GHRLOS *exon sequences. This is a JPEG file showing the comparison of human and putative dog *GHRLOS *exon sequences. The alignments were generated by the ClustalW program and drawn by BOXSHADE . Black shading indicates conserved nucleotides. (A) Exon II (B) Exon 2 (C) Exon 3. Click here for file

Additional file 6 Overview of *GHRLOS *exon 4 and overlapping exons. This is a TIFF file with an overview of *GHRLOS *exon 4 (highlighted in grey) showing the overlap with sense *GHRL *and *SEC13-T *exons (boxed). GT/AG intron splice sites are underlined. The exon 4 polyA signal is depicted in orange font. A stretch of poly(A) in the genomic sequence (exon 4) resulting of frequent oligo(dT) mispriming during cDNA synthesis is highlighted in pink. Click here for file
