# Supplementary figures and images for: Complex organisation and structure of the ghrelin antisense strand gene GHRLOS, a candidate non-coding RNA gene
Source: BMC Mol Biol. 2008 Oct 28;9:95. doi: 10.1186/1471-2199-9-95 (PMC2621237; doi:10.1186/1471-2199-9-95)

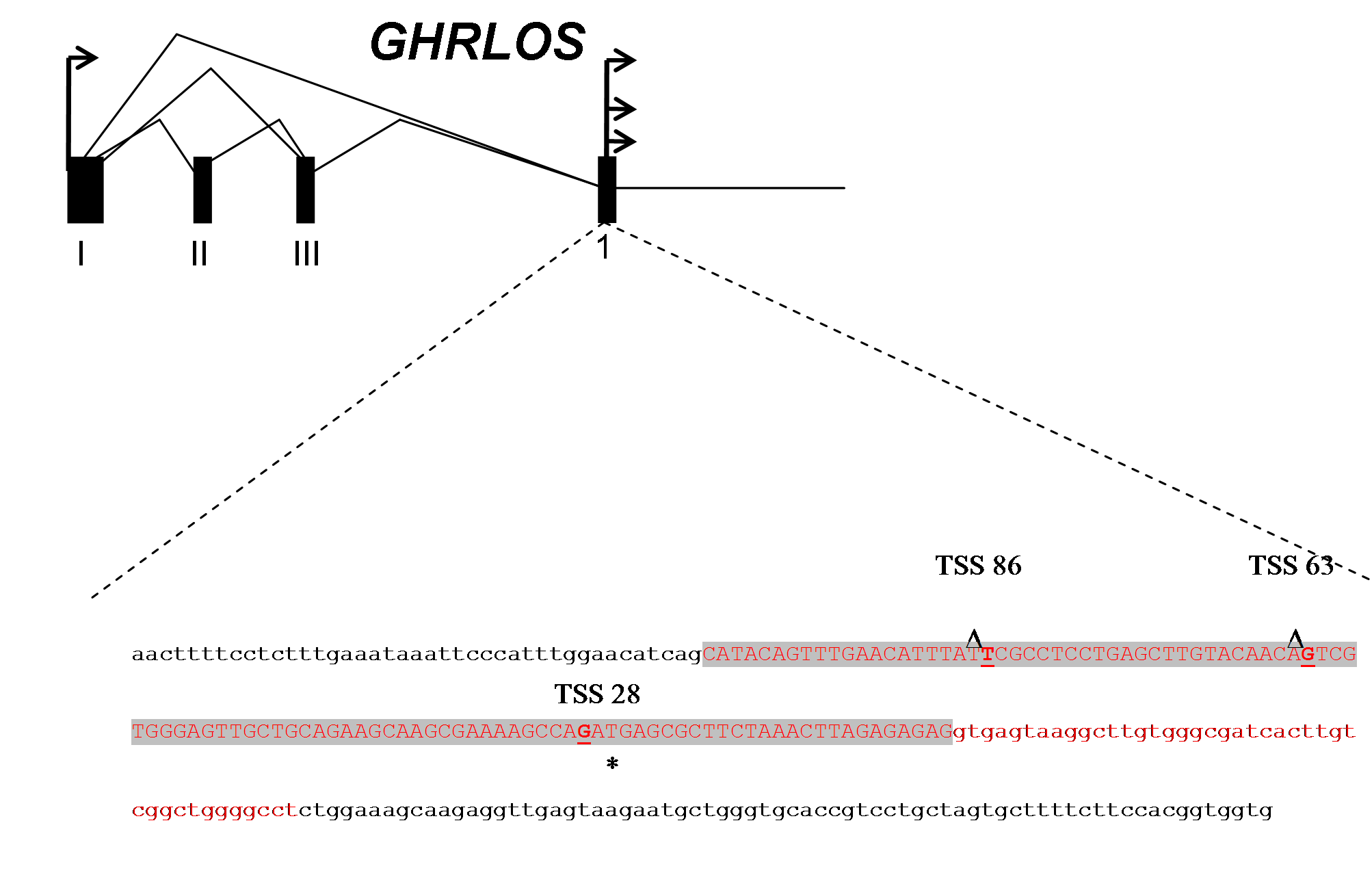

Supplement: Additional file 1 — Overview of GHRLOS exon 1. This is a TIFF file showing GHRLOS transcription start sites and exon 1 sequence. Exon 1 and exons I, II and III, all of which splice into the 106 bp exon 1, are depicted as black boxes. Exons with transcription start sites are indicated by arrows in the direction of transcription. The sequence of the 106 bp exon 1, which splices into upstream exons, is shaded in grey. Transcription start sites in exon 1, previously determined via 5' RACE (Δ) and CAGE (Cap Analysis of Gene Expression) (*), are indicated, and the exact transcription start site nucleotides are underlined and in bold. For comparison, the sequence of the reference (sense) exon 4 of the ghrelin gene [GenBank:NM_016362] is shown in red. GHRLOS exon 1 sequence is shown in upper case, while intron sequence is shown in lower case. [file 1471-2199-9-95-S1.tiff]

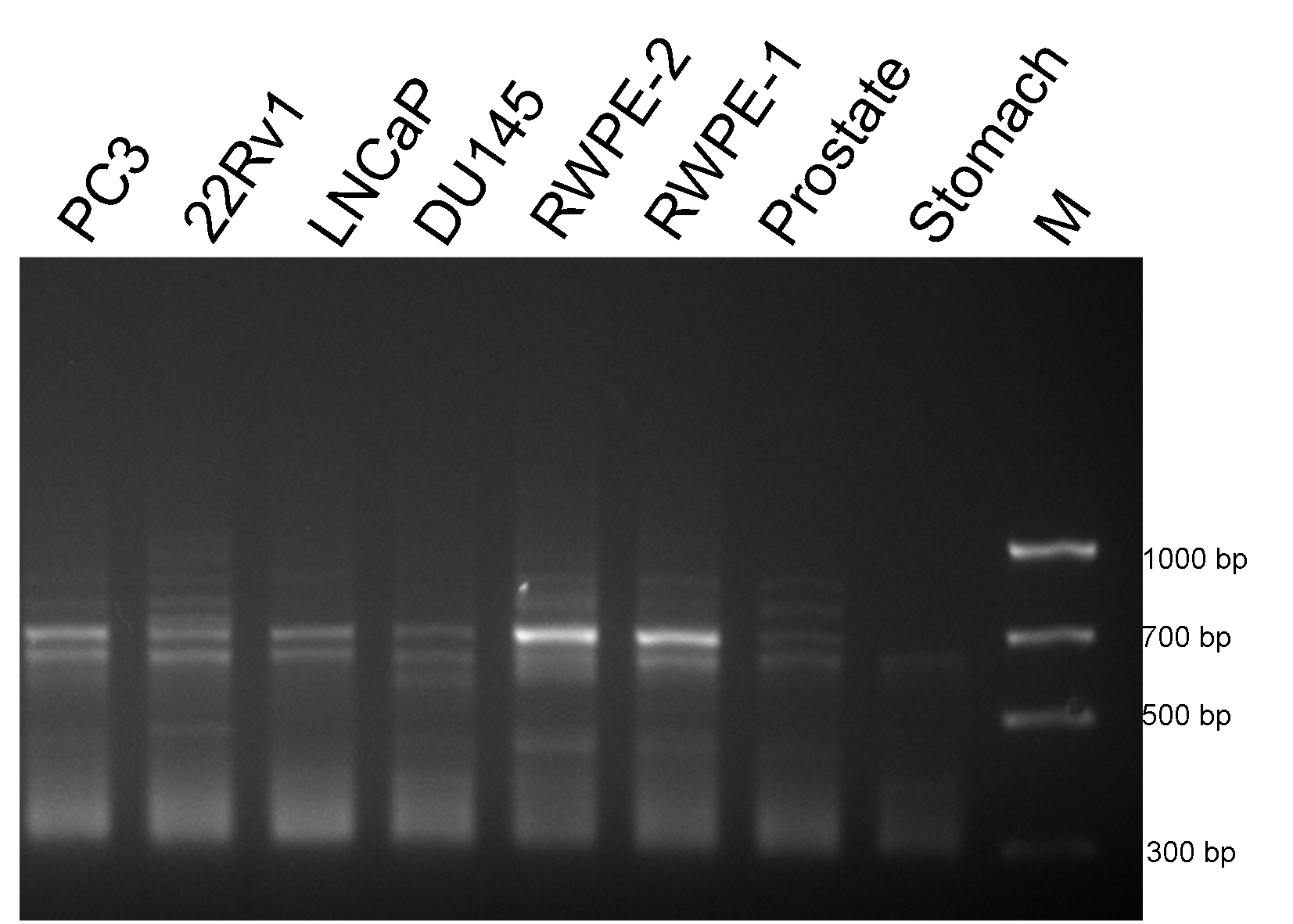

Supplement: Additional file 2 — Ethidium bromide stained agarose gel electrophoresis of GHRLOS exon I a/b to 4 non-quantitative RT-PCR amplicons from cultured cells and normal prostate and stomach tissue. This is a TIFF file showing the expression profile of exon I a/b to 4 amplicons in various human tissues and cell lines, indicating a complex splice pattern. M = MassRuler Express DNA ladder (Fermentas, Burlington, Canada). [file 1471-2199-9-95-S2.tiff]

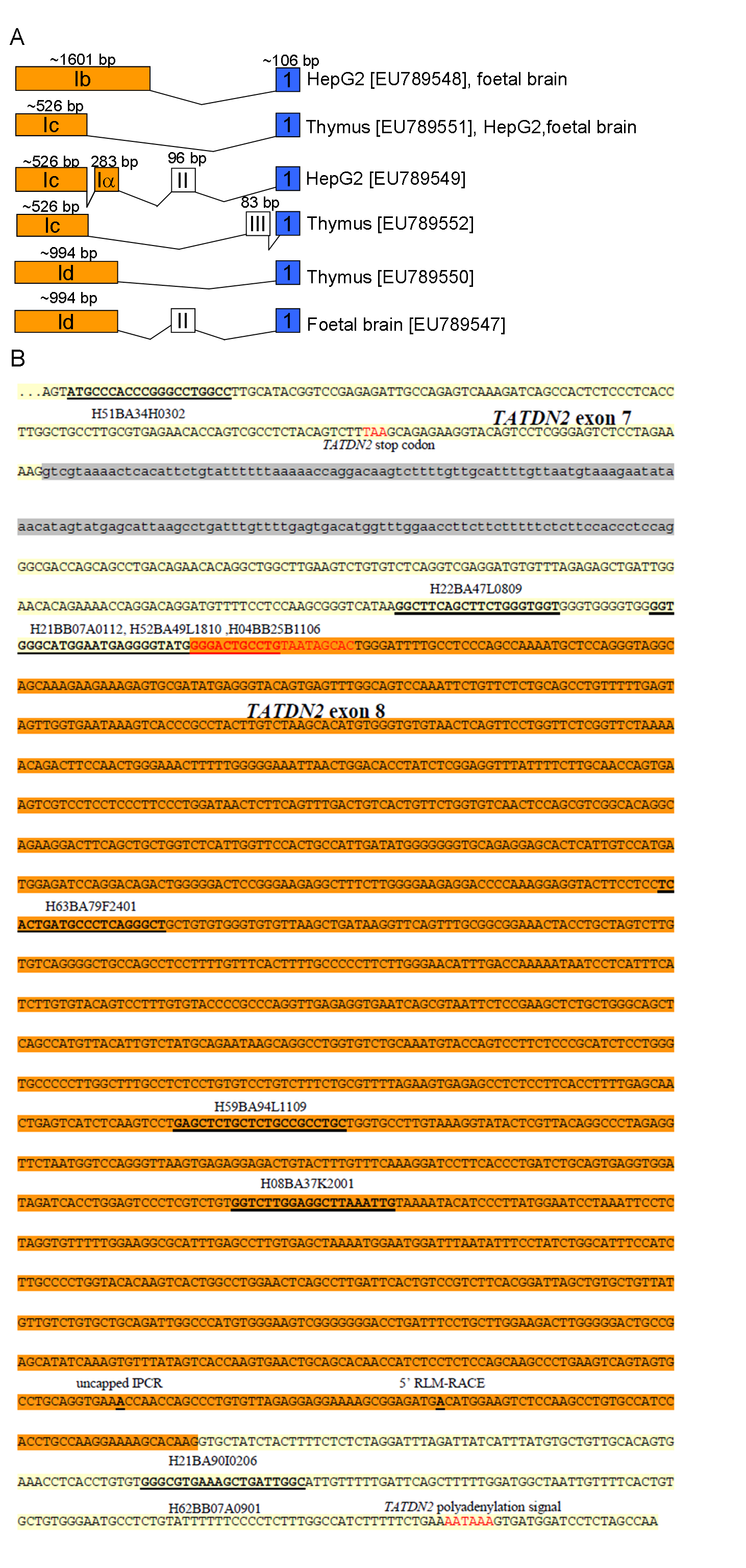

Supplement: Additional file 4 — Overview of CAGE-aided primer walking amplicons and CAGE tags in the 3' untranslated exon 8 of TATDN2. This is a PDF file showing (A) The expression pattern of transcripts spanning exon 8 of TATDN2 (orange) and exon 1 of GHRLOS (blue) revealed via cDNA primer walking (exon shown as boxes, introns as lines, and sizes in bp indicated above each exon). (B) CAGE tags present in the 3' untranslated region of TATDN2 (H51BA34H0302-testis; H22BA47L0809-Hep G2 hepatocarcinoma; H21BB07A0112-Hep G2; H52BA49L1810-adrenal gland; H04BB25B1106-cerebrum; H63BA79F2401-heart; H59BA94L1109-SK-N-AS neuroblastoma cell line; H08BA37K2001-kidney malignancy; H21BA90I0206-HepG2 hepatocellular liver carcinoma; H62BB07A0901-SK-N-AS). TATDN2 exon sequence is shown in beige, intron sequence in lowercase and grey, GHRLOS sequence deduced from cDNA primer walking in orange, and the TATDN2 polyadenylation signal (AATAAA) is shown in red. [file 1471-2199-9-95-S4.tiff]

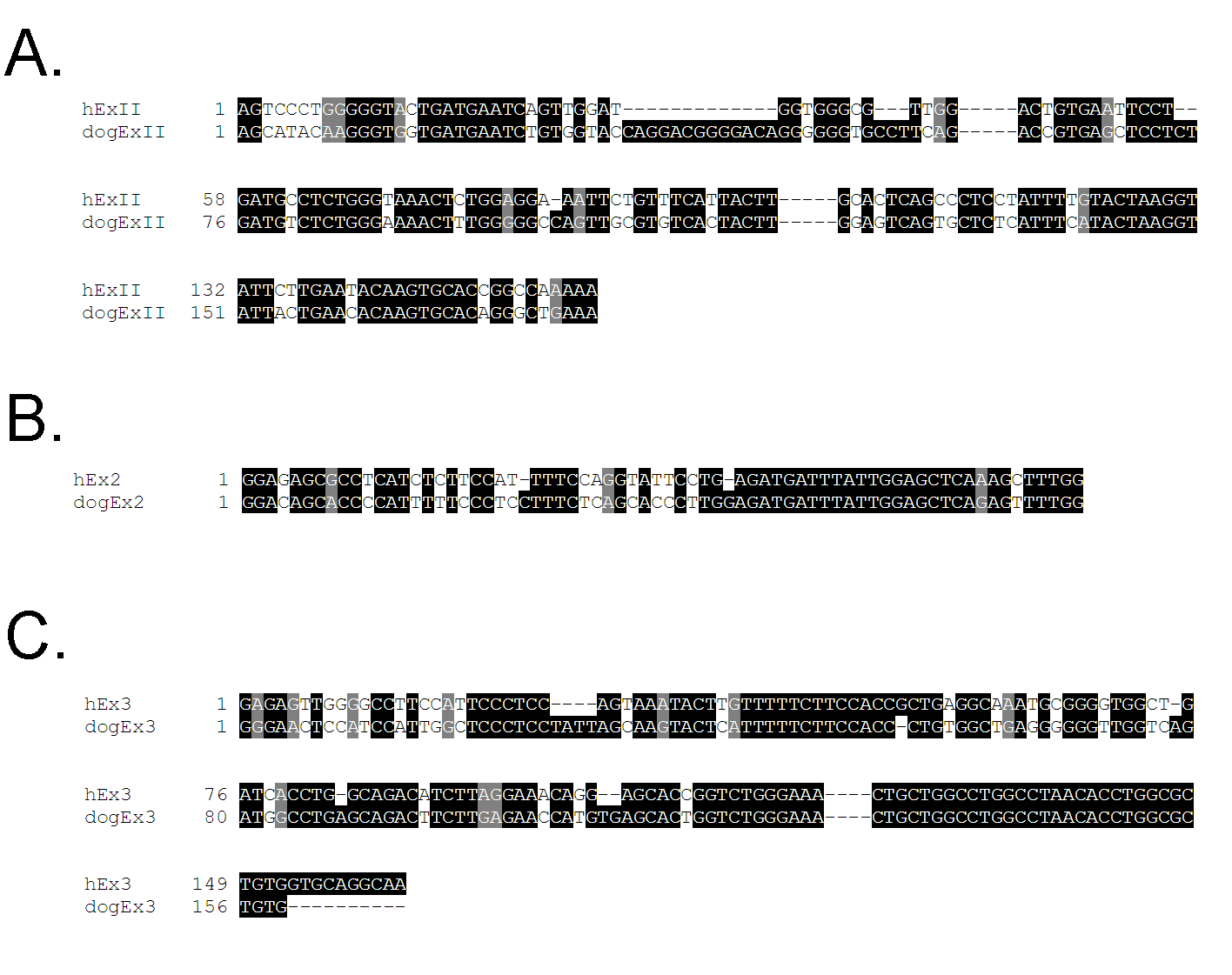

Supplement: Additional file 5 — Comparison of human (h) and putative dog GHRLOS exon sequences. This is a JPEG file showing the comparison of human and putative dog GHRLOS exon sequences. The alignments were generated by the ClustalW program and drawn by BOXSHADE . Black shading indicates conserved nucleotides. (A) Exon II (B) Exon 2 (C) Exon 3. [file 1471-2199-9-95-S5.tiff]

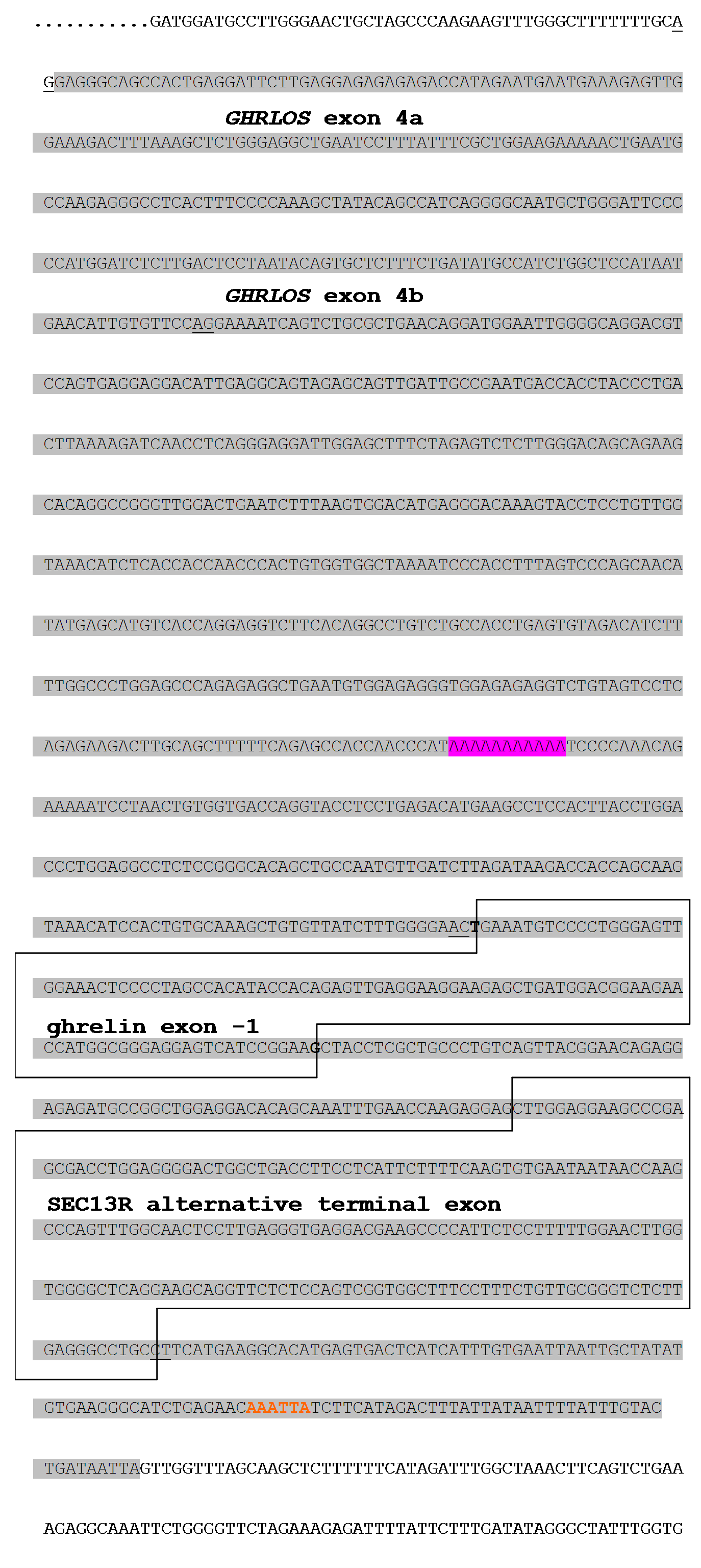

Supplement: Additional file 6 — Overview of GHRLOS exon 4 and overlapping exons. This is a TIFF file with an overview of GHRLOS exon 4 (highlighted in grey) showing the overlap with sense GHRL and SEC13-T exons (boxed). GT/AG intron splice sites are underlined. The exon 4 polyA signal is depicted in orange font. A stretch of poly(A) in the genomic sequence (exon 4) resulting of frequent oligo(dT) mispriming during cDNA synthesis is highlighted in pink. [file 1471-2199-9-95-S6.tiff]
