# Supplementary material for: Complex organisation and structure of the ghrelin antisense strand gene GHRLOS, a candidate non-coding RNA gene
Source: BMC Mol Biol. 2008 Oct 28;9:95. doi: 10.1186/1471-2199-9-95 (PMC2621237; doi:10.1186/1471-2199-9-95)
Supplement: Additional file 3 — Compilation of exons of GHRLOS transcripts. This is a PDF file listing exons of GHRLOS. Exon and intron sizes (bp) are indicated. Experimental evidence from this study and/or external references for each exon are shown. [file 1471-2199-9-95-S3.pdf]

Additional File 4: Compilation of exons of *GHRLOS* transcripts.

This is a PDF file listing exons of *GHRLOS*. Exon and (bp) are indicated. Experimental evidence from this study and/or external references for each exon are shown.

**Exon**	**Size**	**Intron-<br>size (bp)**	**Experimental evidence of external reference**
Ia	51	1138 to exon IIa<br>1138 to exon IIb<br>2520 to exon III<br>4746 to exon 1	TSS: 5' RACE, RT-PCR
Ib	~1601		TSS (CAGE cluster immediately upstream), RT-PCR
Ic	~526	1743 to exon IIb<br>1743 to exon IIb<br>3125 to exon III<br>5332 to exon 1	TSS (CAGE cluster immediately upstream), RT-PCR
Id	~994	185 to exon Iα<br>3593 to exon IIa<br>3593 to exon IIb<br>5820 to exon 1	TSS (CAGE cluster immediately upstream), RT-PCR
Iα	283	1138 to exon IIa<br>1138 to exon IIb<br>2520 to exon III<br>4746 to exon 1	RT-PCR; Splices into 526 bp exon I and 96 bp exon I in the Hep G2 cell line
IIa	157	1225 to exon III<br>3452 to exon 1	RT-PCR
IIb	96	1286 to exon III<br>3513 to exon 1	5' RACE, RT-PCR
III	83	2144 to exon 1	
1a	86	1560 to 2a<br>1434 to 2b<br>2091 to 3a<br>2091 to 3b<br>2149 to 3c<br>2149 to 3d<br>6214 to 4a<br>6469 to 4b	TSS [1]
1b	63		TSS [1]
1c	28		TSS [1]
1d	106		RT-PCR, 5' RACE
2a	68	463 to 3a<br>463 to 3b<br>520 to 3c<br>520 to 3d<br>4586 to 4a<br>4841 to 4b	RT-PCR, [1]
2b	156	501 to 3a<br>501 to 3b<br>558 to 3c<br>558 to 3d<br>4624 to 4a<br>4879 to 4b	RT-PCR
3a	156	3965 to exon 1*a<br>4220 to exon 1*b	RT-PCR, [1]
3b	162		RT-PCR, [1]
3c	101		RT-PCR
3d	105		RT-PCR
4a	~1400	Terminating exon	Terminal exon 3' RACE
4b	~1100	Terminating exon	Terminal exon, 3' RACE

References

1.Seim I, Collet C, Herington AC, Chopin LK: **Revised genomic structure of the human ghrelin gene and identification of novel exons, alternative splice variants and natural antisense transcripts**. *BMC Genomics* 2007, **8**:298.
